# Supplementary material for: Immune Gene Diversity in Archaic and Present-day Humans
Source: Genome Biol Evol. 2018 Dec 19;11(1):232–41. doi: 10.1093/gbe/evy271 (PMC6347564; doi:10.1093/gbe/evy271)
Supplement: Supplementary Data [file evy271_supp.pdf]

Gene diversity in innate immune genes of archaic and present-day humans – Supplementary Material

David Reher*[1], Felix M. Key[1,2,] Aida M. Andrés[1,3⊕], Janet Kelso[1⊕]

[1] Department of Evolutionary Genetics, Max Planck Institute for Evolutionary Anthropology, Leipzig, Germany
[2] Department of Archaeogenetics, Max Planck Institute for the Science of Human History, Jena, Germany
[3] UCL Genetics Institute, University College London, United Kingdom

* Author for Correspondence: David Reher, Department of Evolutionary Genetics, Max Planck Institute for Evolutionary Anthropology, Leipzig, Germany, tel: +49 341 3550 844, david_reher@eva.mpg.de

[⊕] Joint supervision of the project

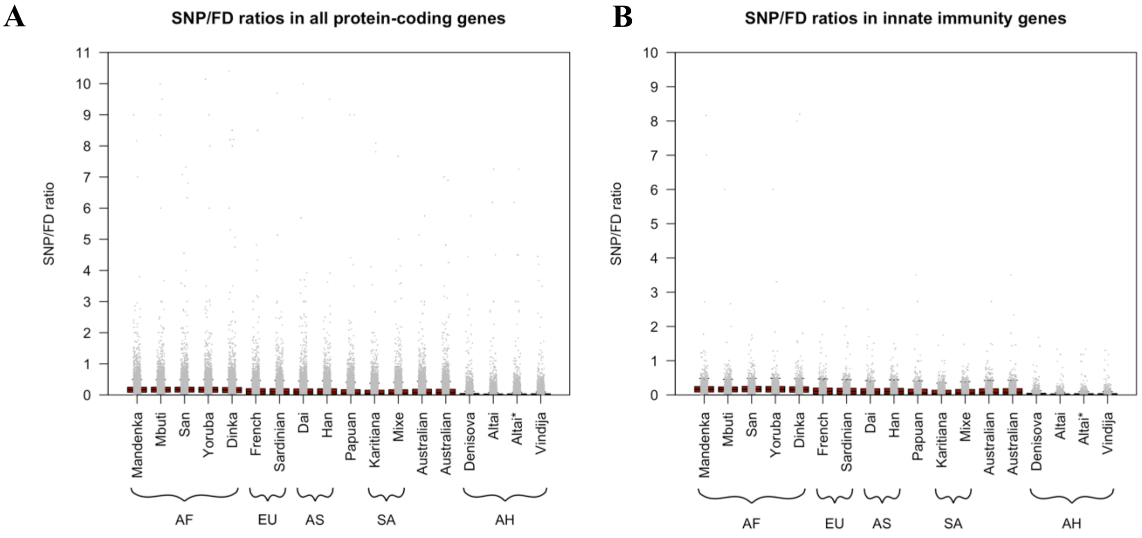

Figure S1: Distributions of SNP/FD ratios per gene for all 17 individuals. Black lines and notches give medians and 95% confidence intervals, respectively. Untrimmed, note differences in scale of the Y-axis. **(A)** All protein-coding genes. Innate immune-related genes. **(B)**. AF = African, EU = European, AS = Asian, SA = South American, AH = Archaic Human.

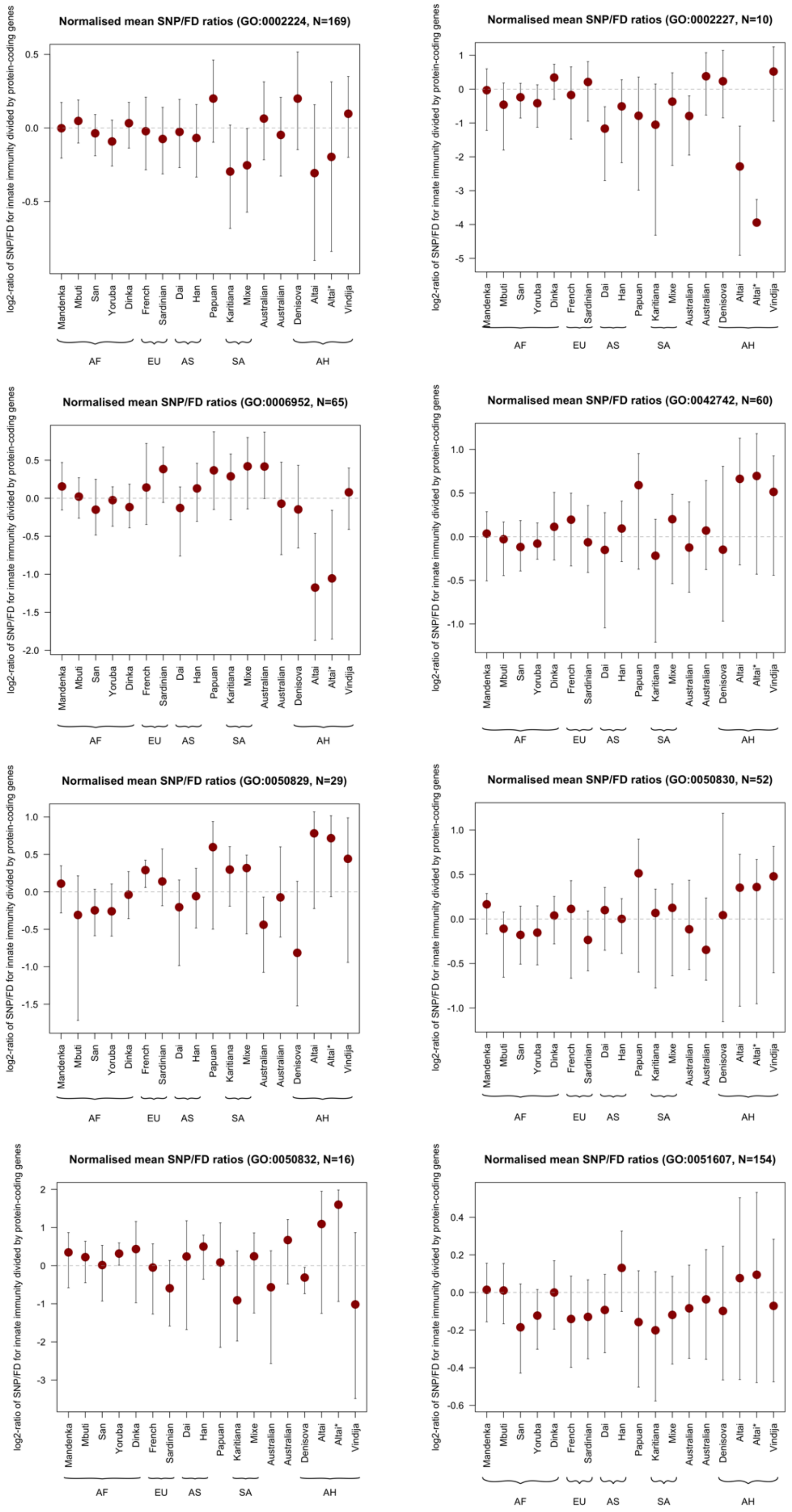

Figure S2: Normalised mean SNP/FD ratios (log2) for all 17 individuals in eight subsets of innate immune-related genes (N from 10 to 169). Error bars give 95% confidence intervals calculated by bootstrapping (B = 5,000). AF = Africa, EU = European, AS = Asian, SA = South American, AH = Archaic Human. Dashed line gives expected value if the mean values for innate immunity genes and autosomal protein-coding background genes were equal. Missing

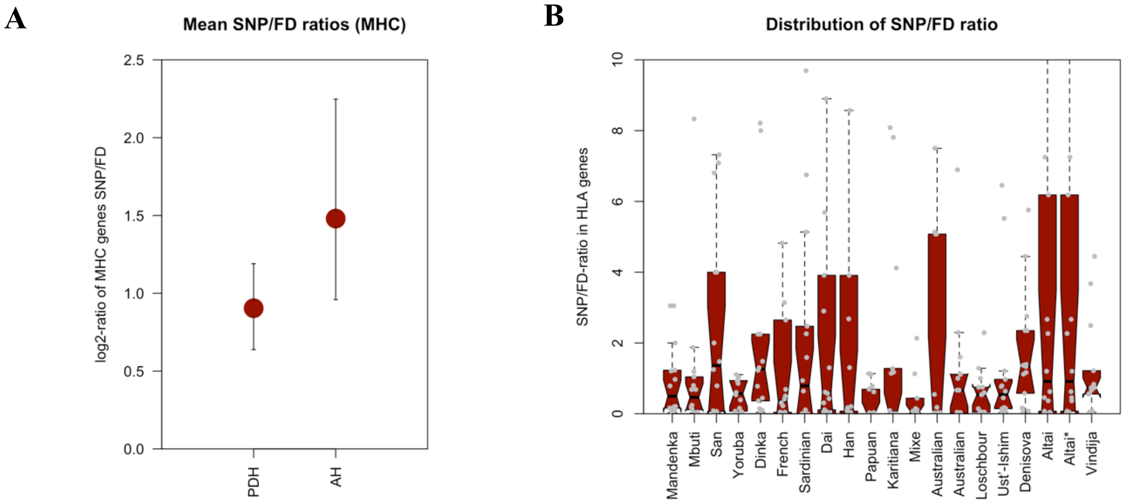

Figure S3: (A) Comparison of mean SNP/FD ratios (log2) of present-day humans (PDH) and archaic humans (AH) without normalisation with protein-coding background genes. Average values for PDH and AH for all MHC genes. Error bars give 95% confidence intervals calculated via bootstrap (B = 5,000). **(B)** Distributions of SNP/FD ratios per MHC gene for all 17 individuals.

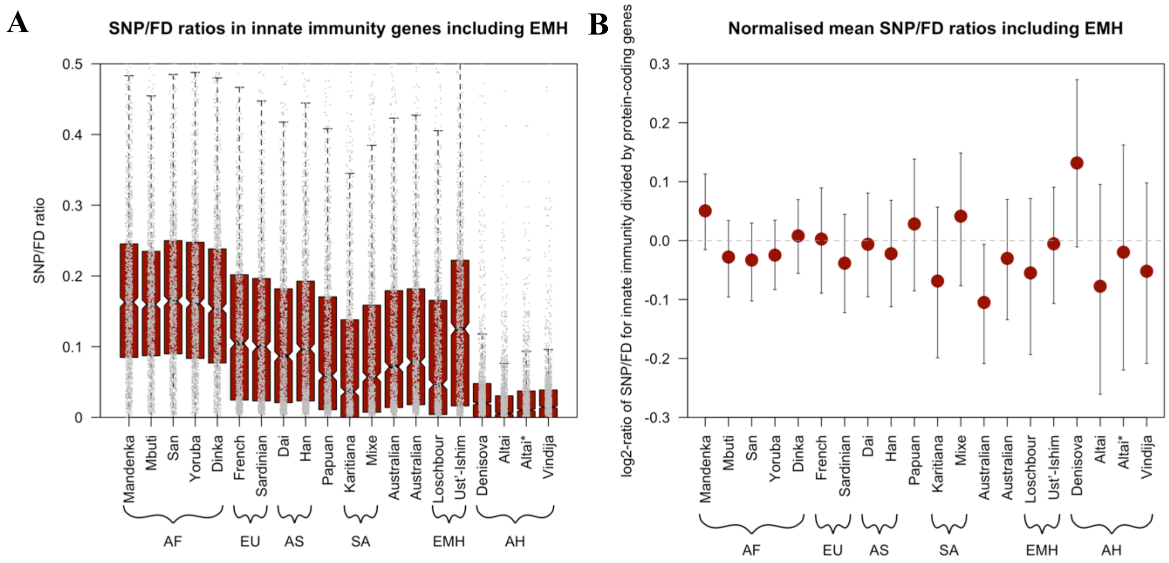

Figure S4: (A) Distributions of SNP/FD ratios per gene in innate immune-related genes (N = 1,548) for all 17 individuals and two early modern humans. Black lines and notches give medians and 95% confidence intervals, respectively. Y-axis trimmed at 0.5 for clarity. **(B)** Normalised mean SNP/FD ratios (log2) for all 17 individuals and two early modern humans in the full set of innate immune-related genes (N = 1,548). Error bars give 95% confidence intervals calculated by bootstrapping (B = 5,000). Dashed line gives expected value if the mean values for innate immunity genes and autosomal protein-coding background genes were equal. AF = African, EU = European, AS = Asian, SA = South American, EMH = Early Modern Human, AH = Archaic Human.

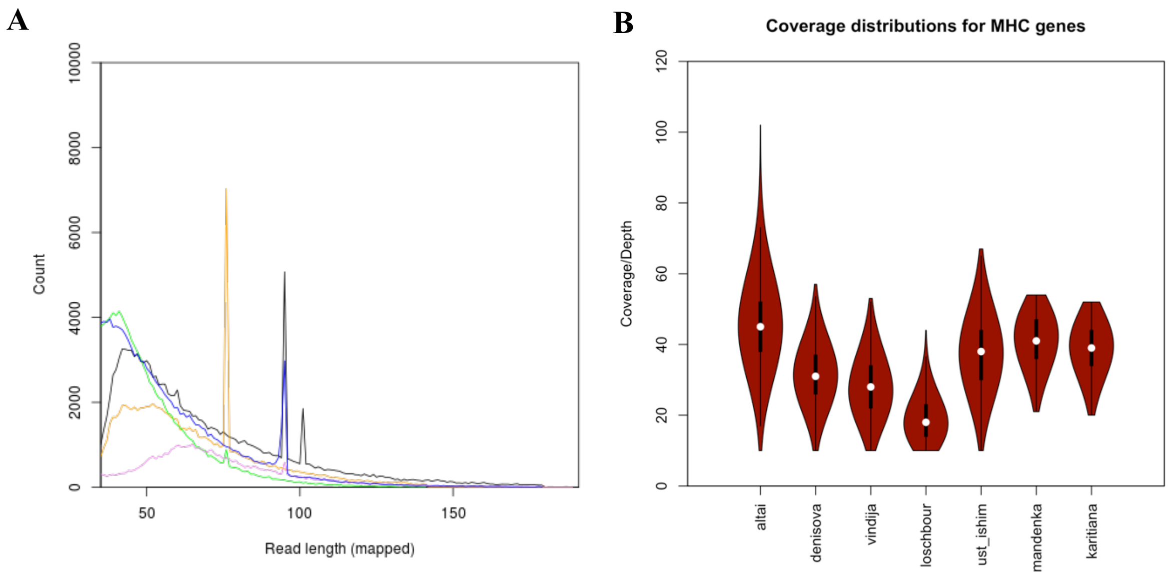

Figure S5: (A) Read length distribution across MHC genes for archaic individuals. Altai (black) and Vindija 33.19 (green) Neandertals, Denisova (orange), Loschbour (pink) and Ust'-Ishim (blue). **(B)** Coverage distributions for MHC genes in the archaic and two present-day individuals.

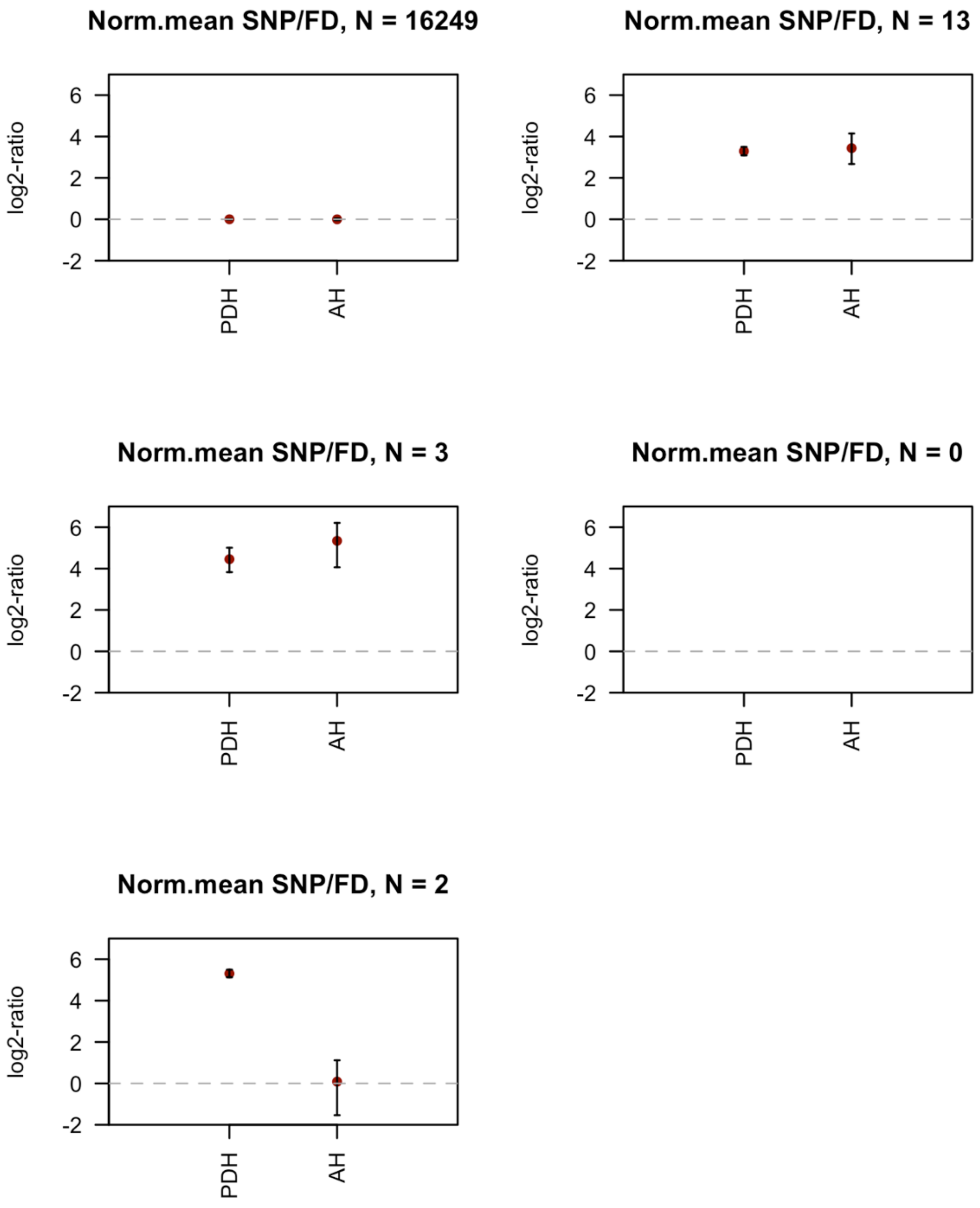

Figure S6: Comparison of SNP/FD ratios in present-day humans to SNP/FD ratios in archaic humans in bins with increasing SNP/FD ratio in present-day humans to investigate if the signal in the MHC genes is driven by general mis-alignments of short ancient reads in regions with high divergence and diversity. In particular, we took genes for which we have data in every present-day individual and averaged the SNP/FD ratios over those 14 individuals. We then defined bins with increasing SNP/FD ratio in present-day humans and compared the SNP/FD ratios in present-day humans to the SNP/FD ratios in the archaics for those genes (which is exactly what we did for the MHC genes). Since the distribution of SNP/FD ratios is strongly skewed towards lower ratios, we considered three types of binning to exclude effects by arbitrary bin definition. Here, bins are defined as five equal-width bins from minimum SNP/FD ratio **(A)** to maximum SNP/FD ratio **(E)**. This has a very skewed distribution: almost all genes are in bin 1, very few in the other bins. There is no obviously higher normalised mean SNP/FD ratio in archaic compared to present-day humans suggesting that the signal is specific to the MHC genes.

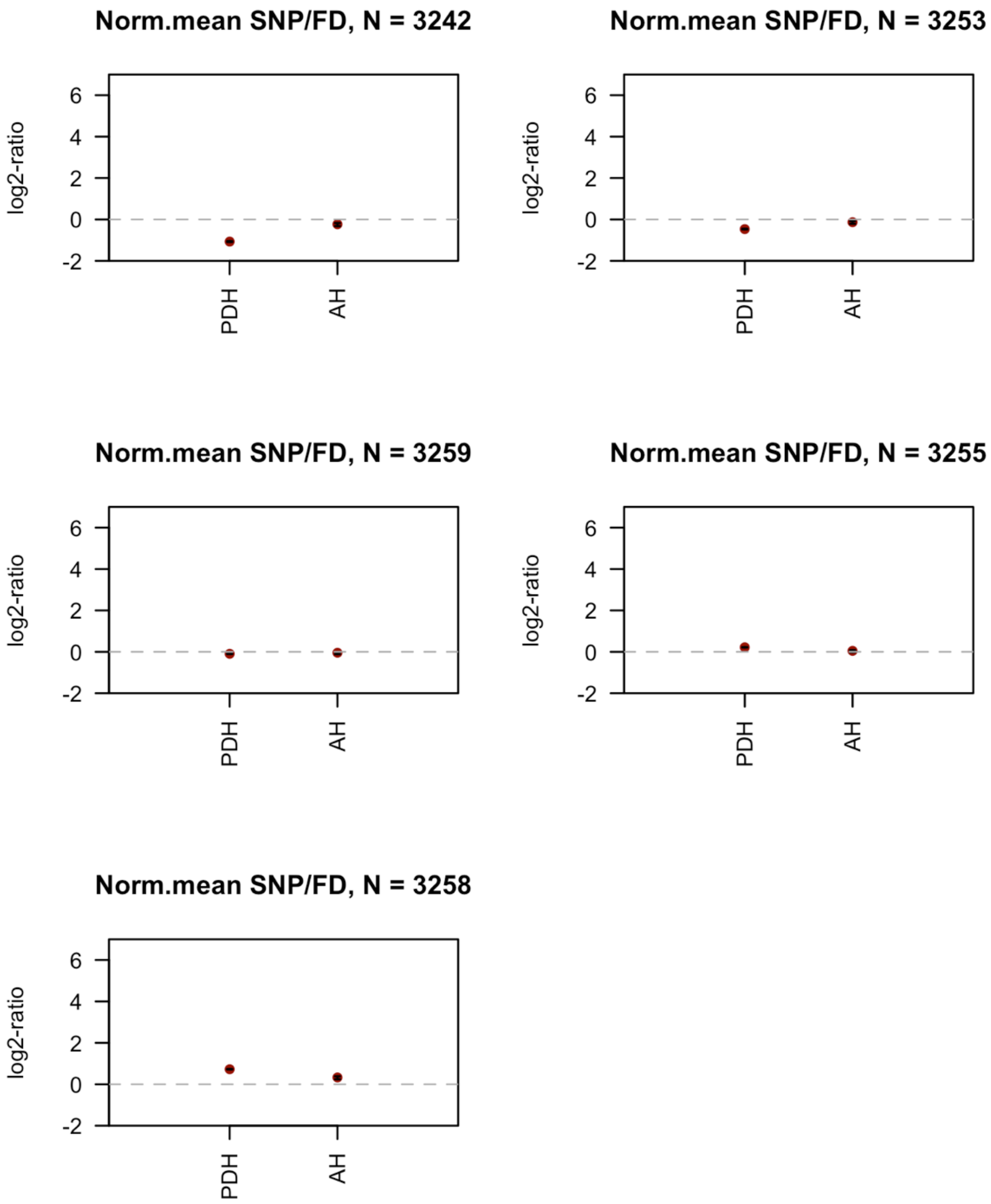

Figure S7: Comparison of SNP/FD ratios in present-day humans to SNP/FD ratios in archaic humans in bins with increasing SNP/FD ratio in present-day humans to investigate if the signal in the MHC genes is driven by general mis-alignments of short ancient reads in regions with high divergence and diversity. In particular, we took genes for which we have data in every present-day individual and averaged the SNP/FD ratios over those 14 individuals. We then defined bins with increasing SNP/FD ratio in present-day humans and compared the SNP/FD ratios in present-day humans to the SNP/FD ratios in the archaics for those genes (which is exactly what we did for the MHC genes). Since the distribution of SNP/FD ratios is strongly skewed towards lower ratios, we considered three types of binning to exclude effects by arbitrary bin definition. Here, bins are defined as five percentile-based bins with approximately equal number of genes in each bin from minimum SNP/FD ratio **(A)** to maximum SNP/FD ratio **(E)** in which the bins do not differ in mean SNP/FD ratios very much. There is no obviously higher normalised mean SNP/FD ratio in archaic compared to present-day humans suggesting that the signal is specific to the MHC genes.

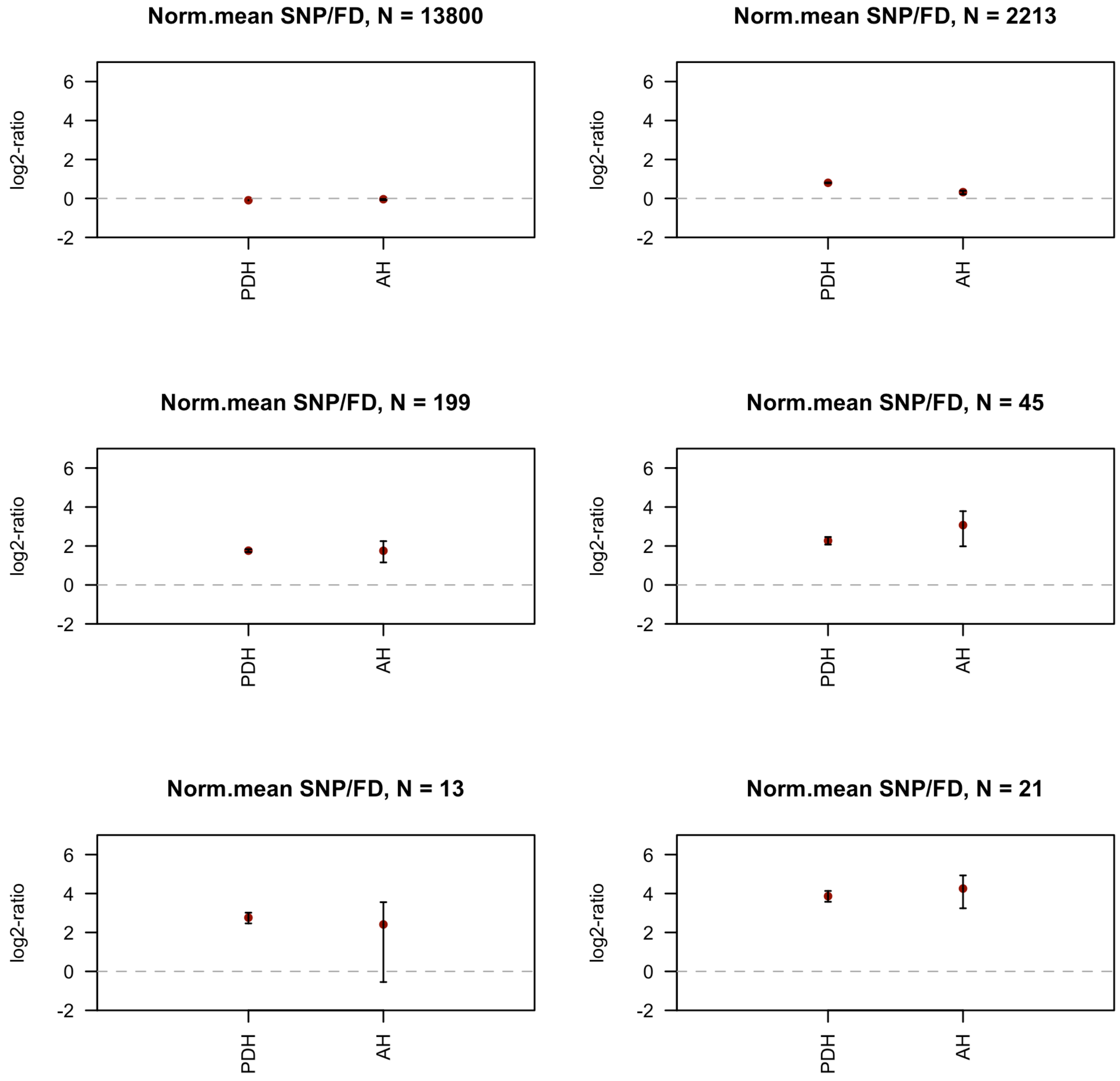

Figure S8: Comparison of SNP/FD ratios in present-day humans to SNP/FD ratios in archaic humans in bins with increasing SNP/FD ratio in present-day humans to investigate if the signal in the MHC genes is driven by general mis-alignments of short ancient reads in regions with high divergence and diversity. In particular, we took genes for which we have data in every present-day individual and averaged the SNP/FD ratios over those 14 individuals. We then defined bins with increasing SNP/FD ratio in present-day humans and compared the SNP/FD ratios in present-day humans to the SNP/FD ratios in the archaics for those genes (which is exactly what we did for the MHC genes). Since the distribution of SNP/FD ratios is strongly skewed towards lower ratios, we considered three types of binning to exclude effects by arbitrary bin definition. Here, bins are defined as five equal-width bins from minimum SNP/FD ratio **(A)** to a SNP/FD ratio of 1 **(E)** and an additional bin for all genes with SNP/FD ratio > 1 **(F)** which decreases the effect of having almost all genes in the first bin with low SNP/FD ratios. There is no obviously higher normalised mean SNP/FD ratio in archaic compared to present-day humans suggesting that the signal is specific to the MHC genes. With this binning, gene set sizes in the high ratio bins are comparable to the MHC region analysis which indicate that if divergence to the reference in the MHC region is a problem it does not seem to affect other genes with very high heterozygosity as much.

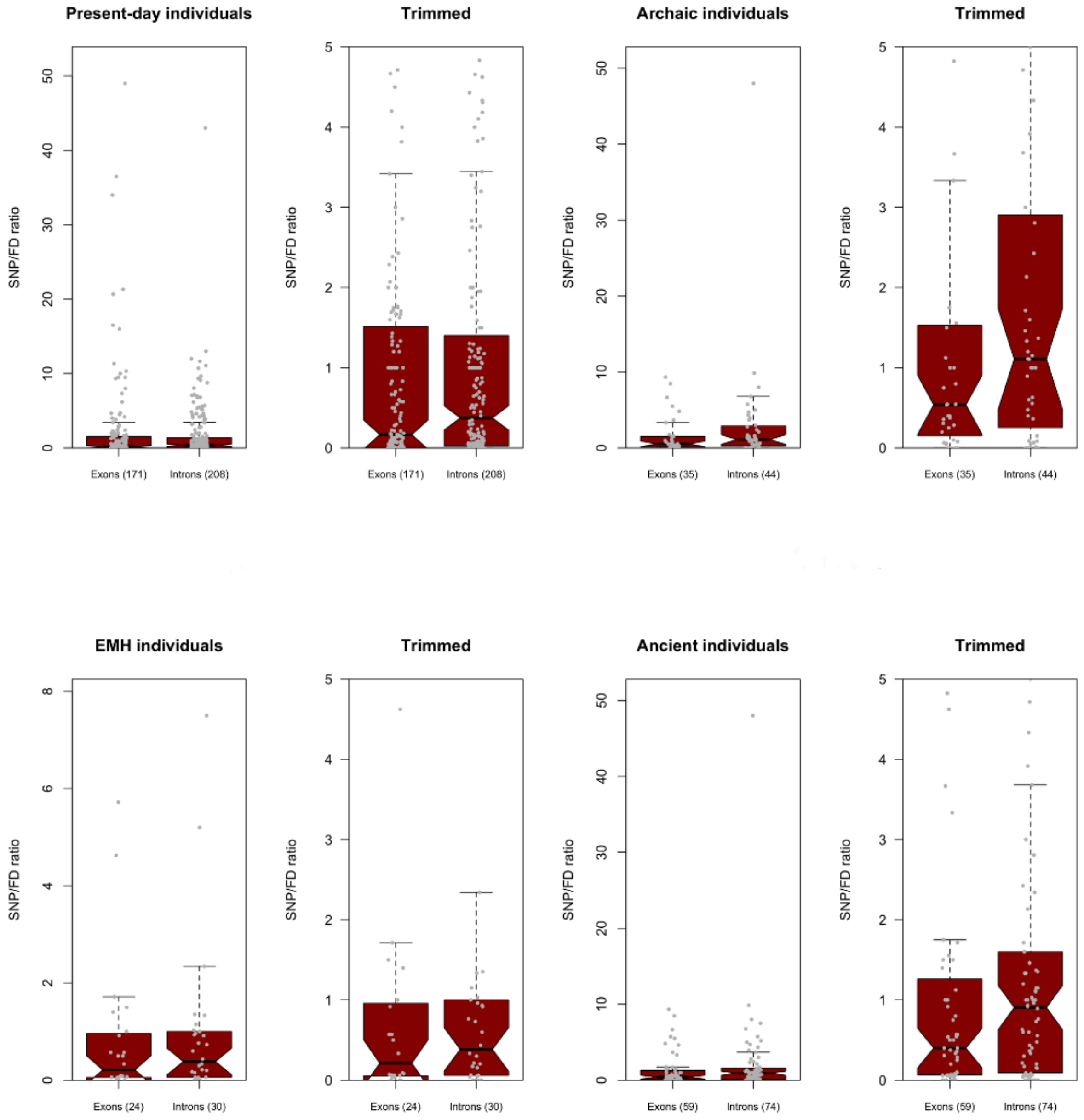

Figure S9: Comparison of SNP/FD ratios in MHC exons versus MHC introns within human groups. None of the comparisons shows significant differences. All comparisons show trends of slightly higher SNP/FD ratios in introns than exons. **(A)** Present-day humans, **(B)** AH = Archaic Humans, **(C)** EMH = Early-modern humans, **(D)** Ancient Humans (AH + EMH).

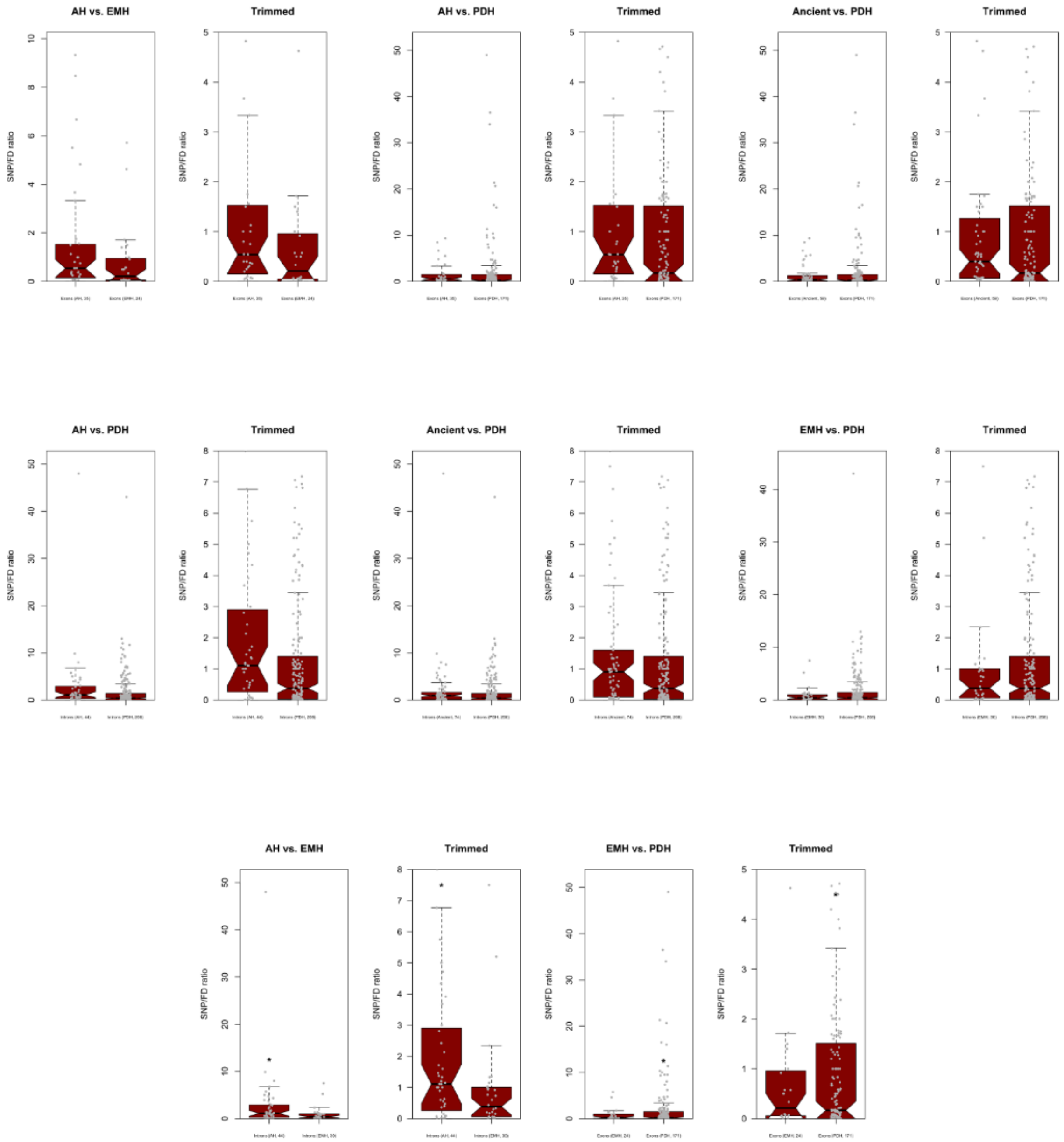

Figure S10: Pairwise comparison of SNP/FD ratios in MHC exons and MHC introns between human groups. **(A)** AH vs. EMH (exons), **(B)** AH vs. EMH (exons), **(C)** AncH vs. PDH (exons), **(D)** AH vs. PDH (introns), **(E)** AncH vs. PDH (introns), **(F)** EMH vs. PDH (introns), **(G)** AH vs. EMH (introns), **(H)** EMH vs. PDH (exons). **(G)** and **(H)** are the only comparisons with nominally significantly different means ($p < 0.05$, no correction for multiple testing) - both show lower SNP/FD ratios in early-modern humans with only two individuals. All other comparisons follow trends observed before: SNP/FD ratios are slightly higher for archaic than anatomically modern humans. PDH = Present-day humans, AH = Archaic Humans, EMH = Early-modern humans, Ancient Humans (AH + EMH)

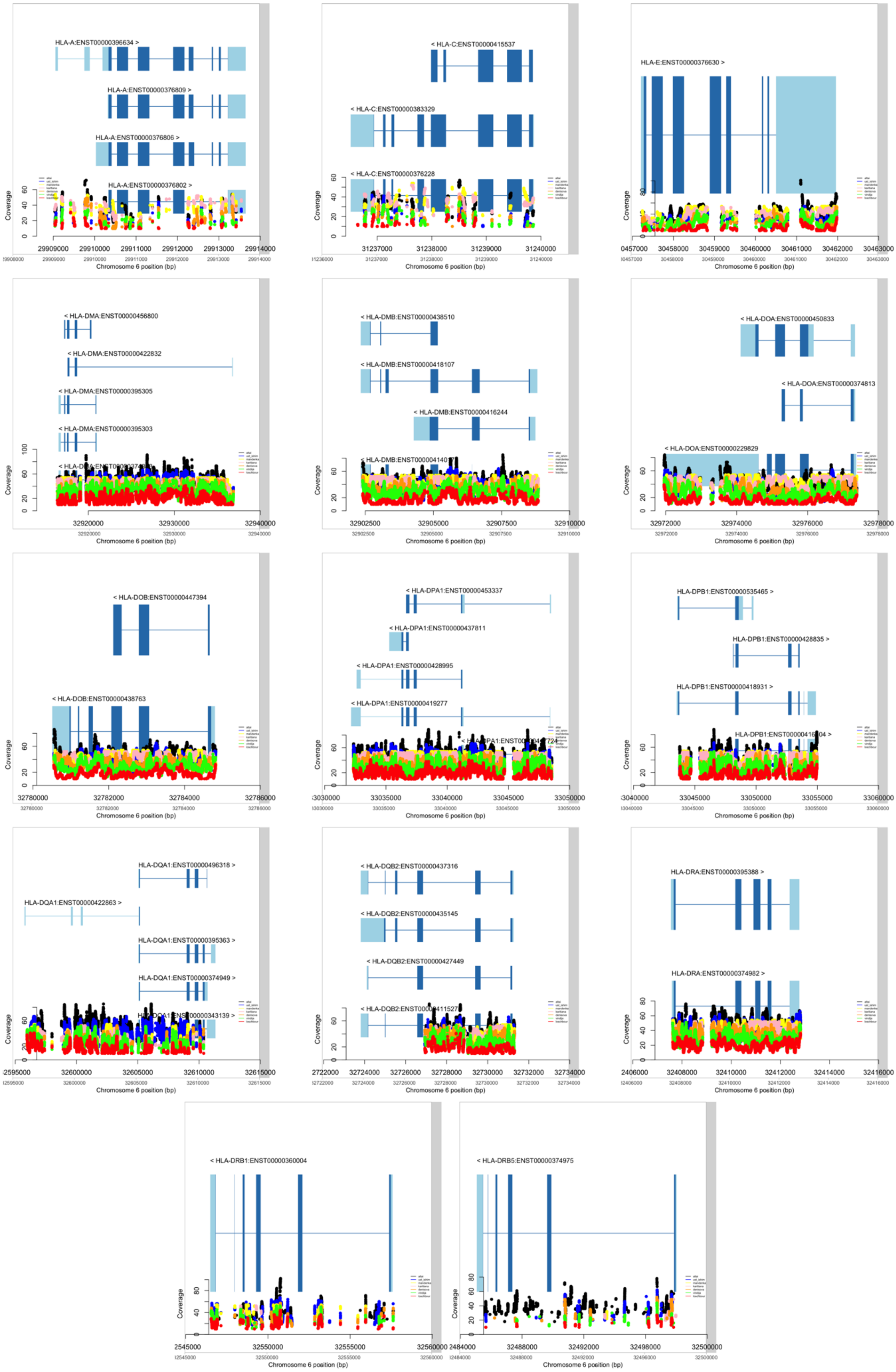

Figure S11: Coverage distribution in MHC genes.

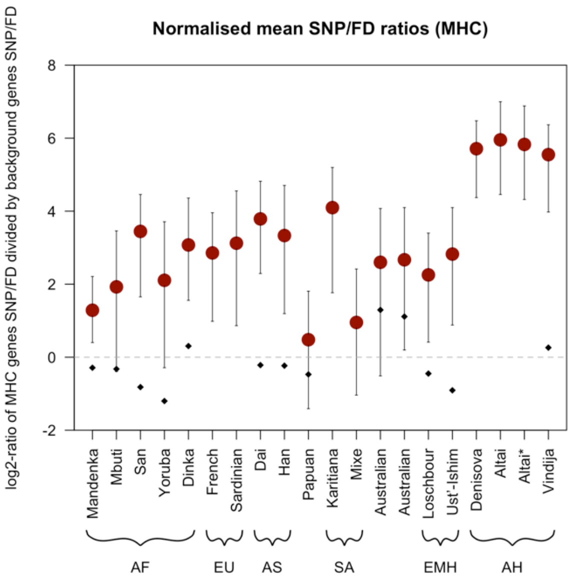

Figure S12: Comparison of normalised mean SNP/FD ratios (log2) of present-day humans (PDH) and archaic humans (AH) including the conserved *B2M* gene (chr15) which is part of the MHC class I genes. Comparison of normalised mean SNP/FD ratios (mean) between single individuals. AF = Africa, EU = European, AS = Asian, SA = South American, EMH = Early anatomically Modern Humans, AH = Archaic Humana. Dashed lines gives expected values if the mean values for innate immunity and autosomal protein-coding background genes are the same.

Table S1: Significantly enriched GO categories among the top 5% tails of the of SNP/FD empirical distribution in two archaic humans (Altai, Denisova) ordered by Family-Wise Error Rate (FWER). FWER values are given after correcting for multiple testing (Bonferroni correction, k = 17).

	**Archaic humans (N = 2)**		
	**GO ID**	**GO Name**	**FWER**
**Top 5%**	0042613	MHC class II protein complex	0
	0032395	MHC class II receptor activity	0
	0004984	Olfactory receptor activity	0
	0042611	MHC protein complex	0.034

Table S2: Significantly enriched GO categories among the top 5% tails of the of SNP/FD empirical distribution in two archaic humans (Altai, Vindija) ordered by Family-Wise Error Rate (FWER). FWER values are given after correcting for multiple testing (Bonferroni correction, k = 17).

	**Archaic humans (N = 2)**		
	**GO ID**	**GO Name**	**FWER**
	0042613	MHC class II protein complex	0
**Top 5%**	0032395	MHC class II receptor activity	0

Table S3: Significantly enriched GO categories among the top 5% tails of the of SNP/FD empirical distribution in two archaic humans (Denisova, Vindija) ordered by Family-Wise Error Rate (FWER). FWER values are given after correcting for multiple testing (Bonferroni correction, k = 17).

	**Archaic humans (N = 2)**		
	**GO ID**	**GO Name**	**FWER**
**Top 5%**	0042613	MHC class II protein complex	0
	0032395	MHC class II receptor activity	0
	0004984	Olfactory receptor activity	0.017
	0042611	MHC protein complex	0.034

Table S4: Significantly enriched GO categories among the top 5% tails of the of SNP/FD empirical distribution in the Denisova ordered by Family-Wise Error Rate (FWER). FWER values are given after correcting for multiple testing (Bonferroni correction, k = 17).

	**Denisova**		
	**GO ID**	**GO Name**	**FWER**
	0042613	MHC class II protein complex	0
**Top 5%**	0004984	Olfactory receptor activity	0
	0042611	MHC protein complex	0
	0032395	MHC class II receptor activity	0
	0004888	Transmembrane signalling receptor activity	0.017
	0004930	G-protein coupled receptor activity	0.017
	0099600	Transmembrane receptor activity	0.034

Table S5: Significantly enriched GO categories among the top 5% tails of the of SNP/FD empirical distribution in the Vindija Neandertal ordered by Family-Wise Error Rate (FWER). FWER values are given after correcting for multiple testing (Bonferroni correction, k = 17).

	**Vindija Neandertal**		
	**GO ID**	**GO Name**	**FWER**
	0042613	MHC class II protein complex	0
**Top 5%**	0032395	MHC class II receptor activity	0
	0004984	Olfactory receptor activity	0.017
	0042611	MHC protein complex	0.034

Table S6: All enriched GO categories among the top 5% tails of the of SNP/FD empirical distribution in the Altai Neandertal ordered by Family-Wise Error Rate (FWER). FWER values are given after correcting for multiple testing (Bonferroni correction, k = 17). Additionally, we show FWER values before correction for multiple testing (FWER*).

	Altai Neandertal			
	**GO ID**	**GO Name**	**FWER**	**FWER***
	0004984	Olfactory receptor activity	0.017	0.001
**Top 5%**	0042613	MHC class II protein complex	0.085	0.005
	0032395	MHC class II receptor activity	0.136	0.008
	0042611	MHC protein complex	0.595	0.035

Table S7: Significantly enriched GO categories among the bottom 5% tails of the of SNP/FD empirical distribution in two archaic humans (Altai, Denisova) ordered by Family-Wise Error Rate (FWER). FWER values are given after correcting for multiple testing (Bonferroni correction, k = 17). GO categories related to virus-related functions are highlighted with bold font.

	**Archaic humans (N = 2)**		
	**GO ID**	**GO Name**	**FWER**
	0004984	Olfactory receptor activity	0
**Bottom 5%**	0004930	G-protein coupled receptor activity	0
	0005125	Cytokine activity	0
	0007186	G-protein coupled receptor signalling pathway	0
	**0019080**	**Viral gene expression**	**0**
	**0019083**	**Viral transcription**	**0**
	0044033	Multi-organism metabolic process	0
	0004888	Transmembrane signalling receptor activity	0
	0099600	Transmembrane receptor activity	0.017

Table S8: Significantly enriched GO categories among the bottom 5% tails of the of SNP/FD empirical distribution in two archaic humans (Altai, Vindija) ordered by Family-Wise Error Rate (FWER). FWER values are given after correcting for multiple testing (Bonferroni correction, k = 17). GO categories related to mitochondria or virus-related functions are highlighted with bold font.

	**Archaic humans (N = 2)**		
	**GO ID**	**GO Name**	**FWER**
	0004984	Olfactory receptor activity	0
**Bottom 5%**	0004930	G-protein coupled receptor activity	0
	0005179	Hormone activity	0
	0044033	Multi-organism metabolic process	0
	**0098800**	**Inner mitochondrial membrane protein complex**	**0**
	**0098798**	**Mitochondrial protein complex**	**0**
	**0019080**	**Viral gene expression**	**0**
	**0019083**	**Viral transcription**	**0**
	0007186	G-protein coupled receptor signalling pathway	0.017
	0005125	Cytokine activity	0.017

Table S9: Significantly enriched GO categories among the bottom 5% tails of the of SNP/FD empirical distribution in two archaic humans (Denisova, Vindija) ordered by Family-Wise Error Rate (FWER). FWER values are given after correcting for multiple testing (Bonferroni correction, k = 17). GO categories related to mitochondrial functions are highlighted with bold font.

	**Archaic humans (N = 2)**		
	**GO ID**	**GO Name**	**FWER**
	0004984	Olfactory receptor activity	0
**Bottom 5%**	0004930	G-protein coupled receptor activity	0
	0004888	Transmembrane signalling receptor activity	0
	**0098800**	**Inner mitochondrial membrane protein complex**	**0**
	0099600	Transmembrane receptor activity	0
	0005125	Cytokine activity	0
	0005179	Hormone activity	0
	**0098798**	**Mitochondrial protein complex**	**0**
	0038023	Signalling receptor activity	0
	0007186	G-protein coupled receptor signalling pathway	0.017
	**0005743**	**Mitochondrial inner membrane**	**0.034**

Table S10: Significantly enriched GO categories among the bottom 5% tails of the of SNP/FD empirical distribution in the Altai Neandertal ordered by Family-Wise Error Rate (FWER). FWER values are given after correcting for multiple testing (Bonferroni correction, k = 17).

	**Altai Neandertal**		
	**GO ID**	**GO Name**	**FWER**
	0004984	Olfactory receptor activity	0
**Bottom 5%**	0005125	Cytokine activity	0
	0004930	G-protein coupled receptor activity	0
	0006415	Translational termination	0
	0006613	Cotranslational protein targeting to membrane	0
	0003735	Structural constituent of ribosome	0
	0022626	Cytosolic ribosome	0
	0044391	Ribosomal subunit	0
	0005840	Ribosome	0
	0005179	Hormone activity	0
	0006614	SRP-dependent cotranslational protein targeting to membrane	0.017
	0006414	Translational elongation	0.017
	0034641	Cellular nitrogen compound metabolic process	0.017
	0090304	Nucleic acid metabolic process	0.034
	0044445	Cytosolic part	0.034

Table S11: Significantly enriched GO categories among the bottom 5% tails of the of SNP/FD empirical distribution in the Denisova ordered by Family-Wise Error Rate (FWER). FWER values are given after correcting for multiple testing (Bonferroni correction, k = 17).

	**Denisova**		
	**GO ID**	**GO Name**	**FWER**
	0004984	Olfactory receptor activity	0
	0004930	G-protein coupled receptor activity	0
	0005125	Cytokine activity	0
**Bottom 5%**	0006412	Translation	0
	0034641	Cellular nitrogen compound metabolic process	0
	0003676	Nucleic acid binding	0
	0003735	Structural constituent of ribosome	0.017
	0005840	Ribosome	0.017
	0030529	Intracellular ribonucleoprotein complex	0.017
	1990904	Ribonucleoprotein complex	0.017
	0044391	Ribosomal subunit	0.017
	0034641	Cellular nitrogen compound metabolic process	0.017
	0006518	Peptide metabolic process	0.034
	0043043	Peptide biosynthetic process	0.034

Table S12: Significantly enriched GO categories among the bottom 5% tails of the of SNP/FD empirical distribution in the Vindija Neandertal ordered by Family-Wise Error Rate (FWER). FWER values are given after correcting for multiple testing (Bonferroni correction, k = 17).

	**Vindija Neandertal**		
	**GO ID**	**GO Name**	**FWER**
**Bottom 5%**	0004984	Olfactory receptor activity	0
	0004930	G-protein coupled receptor activity	0
	0030529	Intracellular ribonucleoprotein complex	0
	1990904	Ribonucleoprotein complex	0
	0005882	Intermediate filament	0.017

Table S13: Non-significantly enriched GO categories **related to mitochondria and virus-related functions only** among the bottom 5% tails of the of SNP/FD empirical distribution in the Altai Neandertal ordered by Family-Wise Error Rate (FWER). FWER values are given after correcting for multiple testing (Bonferroni correction, k = 17). Additionally, we show FWER values before correction for multiple testing (FWER*).

	**Altai Neandertal**			
	**GO ID**	**GO Name**	**FWER**	**FWER***
**Bottom 5%**	0019080	Viral gene expression	0.051	0.003
	0019083	Viral transcription	0.051	0.003
	0005739	Mitochondrion	0.493	0.029
	0098798	Mitochondrial protein complex	0.527	0.031
	0009880	Inner mitochondrial membrane protein complex	0.612	0.036

Table S14: Non-significantly enriched GO categories **related to mitochondrial and virus-related functions only** among the bottom 5% tails of the of SNP/FD empirical distribution in the Denisova ordered by Family-Wise Error Rate (FWER). FWER values are given after correcting for multiple testing (Bonferroni correction, k = 17). Additionally, we show FWER values before correction for multiple testing (FWER*).

	**Denisova**			
	**GO ID**	**GO Name**	**FWER**	**FWER***
	0098800	Inner mitochondrial membrane protein complex	0.068	0.004
**Bottom 5%**	0005739	Mitochondrion	0.085	0.005
	0005743	Mitochondrial inner membrane	0.102	0.006
	0044429	Mitochondrial part	0.17	0.01
	0098798	Mitochondrial protein complex	0.17	0.01
	0005740	Mitochondrial envelope	0.187	0.011
	0019080	Viral gene expression	0.255	0.015
	0019083	Viral transcription	0.255	0.015
	0031966	Mitochondrial membrane	0.306	0.018
	0005761	Mitochondrial ribosome	0.697	0.041

Table S15: Non-significantly enriched GO categories **related to mitochondrial and virus-related functions only** among the bottom 5% tails of the of SNP/FD empirical distribution in the Vindija Neandertal ordered by Family-Wise Error Rate (FWER). FWER values are given after correcting for multiple testing (Bonferroni correction, k = 17). Additionally, we show FWER values before correction for multiple testing (FWER*).

	**Vindija Neandertal**			
	**GO ID**	**GO Name**	**FWER**	**FWER***
	0019080	Viral gene expression	0.153	0.009
**Bottom 5%**	0019083	Viral transcription	0.153	0.009

Table S16: Non-significantly enriched GO categories **related to mitochondria functions only** among the bottom 5% tails of the of SNP/FD empirical distribution in a Mixe individual (SS6004479) ordered by Family-Wise Error Rate (FWER). FWER values are given after correcting for multiple testing (Bonferroni correction, k = 17). Additionally, we show FWER values before correction for multiple testing (FWER*).

	Mixe (SS6004479)			
	**GO ID**	**GO Name**	**FWER**	**FWER***
	0019083	Viral transcription	0.017	0.001
	0019080	Viral gene expression	0.108	0.004
**Bottom 5%**	0098798	Mitochondrial protein complex	0.255	0.015

Table S17: Non-significantly enriched GO categories **related to mitochondria functions only** among the bottom 5% tails of the of SNP/FD empirical distribution in a Han individual (SS6004469) ordered by Family-Wise Error Rate (FWER). FWER values are given after correcting for multiple testing (Bonferroni correction, k = 17). Additionally, we show FWER values before correction for multiple testing (FWER*).

	**Mixe (SS6004479)**			
	**GO ID**	**GO Name**	**FWER**	**FWER***
	0005739	Mitochondrion	0.119	0.007
	0005740	Mitochondrial envelope	0.136	0.008
**Bottom 5%**	0044429	Mitochondrial part	0.238	0.014
	0031966	Mitochondrial membrane	0.765	0.045

Table S18: Non-significantly enriched GO categories **related to mitochondria and virus-related functions only** among the bottom 5% tails of the of SNP/FD empirical distribution in a French individual (SS6004468) ordered by Family-Wise Error Rate (FWER). FWER values are given after correcting for multiple testing (Bonferroni correction, k = 17). Additionally, we show FWER values before correction for multiple testing (FWER*).

	**French (SS6004468)**			
	**GO ID**	**GO Name**	**FWER**	**FWER***
	0019083	Viral transcription	0.272	0.016
**Bottom 5%**	0019080	Viral gene expression	0.306	0.018

Table S19: Non-significantly enriched GO categories **related to mitochondria and virus-related functions only** among the bottom 5% tails of the of SNP/FD empirical distribution in a Papuan individual (SS6004472) ordered by Family-Wise Error Rate (FWER). FWER values are given after correcting for multiple testing (Bonferroni correction, k = 17). Additionally, we show FWER values before correction for multiple testing (FWER*).

	**Papuan (SS6004472)**			
	**GO ID**	**GO Name**	**FWER**	**FWER***
	0019083	Viral transcription	0.595	0.035
**Bottom 5%**	0019080	Viral gene expression	0.68	0.04

Table S20: Non-significantly enriched GO categories **related to mitochondrial and virus-related functions only** among the bottom 5% tails of the of SNP/FD empirical distribution in a Yoruban individual (SS6004475) ordered by Family-Wise Error Rate (FWER). FWER values are given after correcting for multiple testing (Bonferroni correction, k = 17). Additionally, we show FWER values before correction for multiple testing (FWER*).

	**Yoruban (SS6004475)**			
	**GO ID**	**GO Name**	**FWER**	**FWER***
**Bottom 5%**	0019080	Viral gene expression	0.221	0.013
	0019083	Viral transcription	0.221	0.013

Table S21: Non-significantly enriched GO categories **related to mitochondrial and virus-related functions only** among the bottom 5% tails of the of SNP/FD empirical distribution in a Karitiana individual (SS6004476) ordered by Family-Wise Error Rate (FWER). FWER values are given after correcting for multiple testing (Bonferroni correction, k = 17). Additionally, we show FWER values before correction for multiple testing (FWER*).

	Karitiana (SS6004476)			
	**GO ID**	**GO Name**	**FWER**	**FWER***
	0019083	Viral transcription	0.051	0.003
**Bottom 5%**	0019080	Viral gene expression	0.17	0.01

Table S22: Non-significantly enriched GO categories **related to mitochondria and virus-related functions only** among the bottom 5% tails of the of SNP/FD empirical distribution in an Australian individual (SS6004477) ordered by Family-Wise Error Rate (FWER). FWER values are given after correcting for multiple testing (Bonferroni correction, k = 17). Additionally, we show FWER values before correction for multiple testing (FWER*).

	**Australian (SS6004477)**			
	**GO ID**	**GO Name**	**FWER**	**FWER***
**Bottom 5%**	0098800	Inner mitochondrial membrane protein complex	0.34	0.02
	0098798	Mitochondrial protein complex	0.357	0.021
